# Models of Housing in the Quebec Setting for Individuals With Mental Illness

**DOI:** 10.3389/fpsyt.2019.00850

**Published:** 2019-12-20

**Authors:** Henri Dorvil, Julien Tousignant-Groulx

**Affiliations:** ^1^School of Social Work, University of Quebec at Montreal, Montreal, QC, Canada; ^2^University of Quebec at Montreal, Montreal, QC, Canada; ^3^Research Center, Montreal Mental Health University Institute, University of Montreal, Montreal, QC, Canada; ^4^Department of Psychology, University of Quebec at Montreal, Montreal, QC, Canada; ^5^Psychology Lab on Health and Quality of Life, University of Quebec at Montreal, Montreal, QC, Canada

**Keywords:** housing, Quebec (Canada), mental illness, supported housing, supportive housing, permanent supportive housing (PSH), social integration, rehabilitation

## Abstract

The World Health Organization (WHO) defines mental health as “a state of well-being in which every individual realizes his or her own potential, can cope with the normal stresses of life, can work productively and fruitfully, and is able to make a contribution to her or his community”. A person’s mental health is shaped by various social, economic, physical, and environmental factors, at different stages of life. Risk factors are heavily associated with social inequalities in the domains of employment, housing, and education. Theories of social determinants of health postulate the beneficial effects of factors exterior to medicine (regarding income, housing, education, and employment) on the health of individuals and populations. Recognition of the effect of social determinants on the health of vulnerable populations has been at the core of the intervention models and housing services developed by social service professionals in Québec. This article offers a review of housing services provided to psychiatric patients living in the community, over the last 50 years in Quebec. Different models of housing with social support which contribute to the autonomy, the security, and the empowerment of psychiatric patients are presented.

## Housing for Mentally Ill People in Quebec: a Historical Perspective From 1970 to 2020

Housing affects every aspect of one’s life and influences the environment in which an individual develops itself. The lack of adequate housing can notably impact the access to education, work, or basic amenities such as security, water, and food (1), which have a documented impact on health (2–4). It plays an important role in social integration and is a pillar of a functioning society for all. In that regard, the Universal Declaration of Human Rights declared housing as a fundamental right in 1948: “Everyone has the right to a standard of living adequate for the well-being of himself and of his family, including food, clothing, housing, and medical care and necessary social services…” (Article 25.1) (5). Housing has also been found to be a crucial determinant of mental health (6). However, as Dorvil et al. (7) mention, mental health policies of the past understated the importance of suitable housing as a factor for recovery.

Although many individuals living with mental illness still live in their naturally occurring network (see section on different housing models), lack of affordable housing leaves many in poor conditions which limits their ability to recover and to be independent. This leads to increased healthcare costs for the state, since many individuals receiving services in hospitals could be helped more efficiently by community services (6). The Mental Health Commission of Canada (6) has found that 520,700 Canadians with mental illness do not have access to adequate housing and reports that up to 119,800 of them are homeless.

Prior to the deinstitutionalization of housing services in Quebec, most people with severe mental illness were housed in institutional facilities, or asylums (8) with poor living conditions that perpetuated the segregation of people with mental illness and further increased the patients’ social isolation (9). Many of these institutional wards were overcrowded and did not offer much opportunity for patients to rehabilitate, which led to an increasing number of individuals living in poor conditions. The release of these patients in the community, following significant mental health policy changes in the 1960s in Quebec led to a considerable reorganization of housing services and treatment of individuals with mental illness (7).

The *Réseau Québecois des OSBL d’Habitation* (10) supports that deinstitutionalization in Quebec was separated in three different processes. De-hospitalization, the first process achieved, resulted in a massive exodus of mental health patients from psychiatric institutions. From 1965 to 1981, there was a 70% reduction of inpatients in mental hospitals (11). From 1960 to 2002, the Louis-H. Lafontaine hospital, the biggest mental health hospital in the province, reduced its occupancy from 7,500 to 700 (9). These considerable changes brought by deinstitutionalization created an important need for housing throughout the community. The second and third processes, non-hospitalization (reducing the dependency of people with mental illness on hospitalization for treatment) and healthcare system reorganization (redistribution of services in the community and social reinsertion), were accessed at a much slower pace (10). While some progress initially highlighted the need for community-based services for individuals with mental illness (12, 13), reforms to the healthcare system led by Claude Castonguay in 1971 limited governmental implication in community-based services (10). In 1989, however, the Mental Health Policy report from the Quebec Ministry of Health and Social Services (MSSS—Ministère de la Santé et des Services Sociaux) supported the necessity for institutions and government policies to develop and fund community-based services that allowed long-term treatment and rehabilitation of people with mental illness (14). MSSS also identified housing, work and quality of services as priorities for the reintegration of individuals with mental illness in 1998 (15). Further deinstitutionalization of these services was planned between 1997 and 2002, as 3,000 patients were to be removed from psychiatric hospitals and redistributed into community housing (16).

MSSS renewed their mission concerning mental health and housing in their 2005 Mental Health Action Plan (17). The plan included financial support for housing available for individuals with mental illness. The report suggests that autonomous housing with support options are insufficient with 491 rooms (compared to other options such as intermediary resources with 2,967 rooms and family housing, which houses 4,385 individuals) and that more options should be available to these individuals so that they can choose housing that corresponds to their needs and that promotes social integration.

In the 2015 Mental Health Action Plan, MSSS restates its mission to reorganize the housing resources available to mental health patients by providing more rent supplements for individuals in need. In addition to services already offered, at least 10% of all housing options offered by the *AccèsLogis* program, plus 500 initial places were to be reserved for homeless individuals or individuals with mental illness (18).

The development of adequate community housing was by no means immediate and was not as sudden as the deinstitutionalization of these services. In fact, at first, deinstitutionalization led to increased homelessness and incarcerations due to poor planning related to community services (6). In the last 50 years, housing models for people with mental illness or other marginalized individuals evolved considerably through trial and error and research (see section on housing models below). Dorvil et al. (7) still note that: “However, public, community, and social housing resources are still insufficient to accommodate this de-institutionalized population. There is a high occupancy rate, and waiting lists are very long.” (p. 499).

The rest of this article is separated in four sections. The first presents and describes different housing models from the first ones put in place after deinstitutionalization until today. The second section describes the findings of Dorvil et al. (7) study on the qualitative effects of these different housing models on its users. The third section is dedicated to the influential *At Home/Chez Soi* project and its repercussions on housing for marginalized groups in Quebec. The fourth section presents permanent housing with support, a current model that is gaining traction in Quebec.

## Different Housing Models

Defining the different services offered following the massive deinstitutionalization of housing for people with mental illness allows for a deeper understanding of their evolution in the last 50 years. Dorvil et al. (7) conducted a qualitative study on the subjective effects of different housing models on residents in Quebec. The study presented four different housing models, in addition to the family homes model (living with one’s family).

### The Family Home Model

As the de-institutionalization of psychiatric patients is a current issue in the field, it is important to recognize that an estimated 70 percent of people with psychiatric disorders live with their families (19–24). It is a simple solution to the housing problem, since these patients stay in their naturally occurring network and do not require state funds to function. However, we argue that this model is not sufficient for the rehabilitation of these patients. Caregiving is especially stressful for the families, as they are rarely prepared to deal with the onset of the disorders that can be accompanied with stressful behavior (25). For example, the severity of negative symptoms of schizophrenic patients was found to be correlated to the objective and subjective caregiver burden of their relatives (25). In addition to this, mental illness patients’ family members deal with constant stress caused by discrimination, lack of services, and lack of understanding related to their family member’s struggles (26). Caregiving in these conditions requires constant energy and can put a strain on the family members’ relationships (26) and can impair family functioning (27). Lack of social support has also been found to be associated with the onset of depression in family caregivers (27). This is especially problematic when individuals with psychiatric disorders are likelier to relapse in tense environments (28). This model requires stronger services and support from external organizations for the patient and the family itself to reduce their burden. Customized services that acknowledge the crucial role of these family members in recovery, while offering support when necessary, are essential to help these families overcome the difficulties associated to family housing. More research and services, such as psychoeducation, are required to allow mental health care professionals to offer better support and promote resiliency in families living with a mentally ill relative (27). In turn, these improvements could potentially reduce re-admissions in psychiatry (9).

### The Custodial Model

This model offers long-term residential accommodations (foster homes) in which services are offered by non-professionals (9) as an alternative to the institutionalization of patients. However, these accommodations tend to perpetuate the problems associated to institutionalization and the retention of behavioral problems while failing to provide the required support to facilitate recovery (29, 30). Recently however, MSSS has recognized the burden of care of these homes and offers financial compensations to help alleviate this burden (31). In addition to this, direct services and training (e.g. psychoeducation on the individual’s mental illness) are offered to better support these caregivers. Crisis centers are also available for patients when necessary.

### Supportive Housing Model

This model is the natural progression from the previous model. It is intended as a professional therapeutic residential accommodation and is based on rehabilitation and skill development values. The end goal of this model is to allow residents to develop their own abilities and live autonomously (32). However, studies have shown that residents of this model do not tend to move further in the continuum of housing models and most stay in these accommodations (33, 34). Housing being conditional to receiving treatment is another criticized aspect of this model (35). This has the consequence of leaving some individuals with mental illness not “housing-ready” and does not access the high rates of homelessness found in these individuals (36, 37).

### Supported Housing Model

This model is an answer to the problems of the previous model. There is a clear distinction between housing and treatment. Patients are encouraged to find housing (usually private apartments) themselves and then receive adapted support on site. The model aims at developing the patients’ autonomy and promotes empowerment while offering long-term support. This model also values housing as a right and as a prerequisite for effective rehabilitation and values naturally occurring support as a means to rehabilitation (38). The distinction between supported and supportive housing models is however not so clear-cut in the literature. While some do operationalize their differences, many authors use the two terms interchangeably (38). The theoretical distinction between these models is however relevant to their historical analysis.

### One-room Housing Model (Autonomous Housing)

This model accounts for the many service-users who live in private or subsidized autonomous one-room housing. This model is not under governmental control and is devoid of any form of control or standards. The repercussion of living in such housing on service-users is relatively unknown and support services are rarely offered on-site.

## The Qualitative Efficacy of Housing Categories in Quebec

While these different models historically succeed each other, their application does not, as services offered in Quebec are varied. However, In their study, Dorvil et al. (9) separate these different models into two categories: the residential accommodations (custodial and supportive models) and the apartment-type resources (supported and autonomous housing). Residential accommodations referred to settings where length of stay was limited, active rehabilitation took place, and day to day activities were supervised by staff. Apartment-type resources referred to settings where no limit was established for the length of stay and where housing was not associated with active rehabilitation.

Generally, younger participants preferred non-structured environments compared to older participants who preferred structure. This is potentially a consequence of long-term hospitalization, which fewer younger participants undergo. The continuum of housing options lead to a perceived hierarchy, where participants “moved up” in the system as their autonomy increased. This perception was also coupled with a perceived hierarchy among the residents of a same housing facility, where socialization and social status are a determining factor.

The residential accommodations seemed superior to apartment-type resources for the management and coping associated to one’s illness. These accommodations offered more services and social interaction possibilities. Staff helped residents with their medication, which residents found especially useful, since they relied on medication to control their illness. Being around other people living with similar difficulties helped the residents by offering them a safety net and a circle of care. Participants in these accommodations sacrificed their autonomy, but were better protected from loneliness, which participants mentioned as a cause for relapse. The fact that residents did not have to conceal their illness helped further reduce the stigmatization associated to the illness and offered a safe place for participants to develop their own abilities. In apartment-type resources, participants often hid their illness and felt scared by the judgement of others, which lead to more isolation. Residential accommodations offered better opportunities than apartment-type resources for self-development. These accommodations are explicitly based on principles of self-development and rehabilitation. Apartment-type resources offered better opportunities for the development of individuality and autonomy, as its residents took their own decisions. Although some support was available to residents from the apartment-type resources, these services were less available than in residential accommodations and residents must further rely on their own judgement.

The opportunity to have personal space to withdraw to was especially beneficial to participants. In that regard apartment-type resources offered better opportunities for its residents to have their own space, since their apartment was private and was not shared with other residents. This also allowed them to have an active sexual life, which the lack of intimacy in residential accommodations prevented. Shared space is common in these accommodations, and residents rarely have more than one room to call their own. Participants living in apartment-type resources had more opportunities to personalize their space and had full control over their own schedule. Residential accommodations limited this by having tight schedules (e.g. curfews) and limiting one’s ability to customize its space. These restraints were generally perceived as excessive control by its residents.

Residential accommodations offered better opportunities to socialize and interact with other people compared to apartment-type resources. These accommodations offered many social activities and promoted interactions between residents. Social skills and problem solving were encouraged and monitored by professionals. Participants who lived in apartment-type resources had to develop their social network outside from their home, but often depended on relationships they established prior to their residency (e.g. people they met during hospitalization). Loneliness was a recurrent problem associated with living in apartment-type resources.

Financial security was perceived as a constant worry by the participants, especially considering their low income. Residential accommodations offered better work and financial opportunities to its residents. Workplace integration programs were often included in these accommodations and its skill development opportunities led to easier employability. Staff from those facilities also helped residents in their budget management and were useful resources for interactions with welfare agents (as welfare was the principal source of income for most participants). These accommodations were also generally less expensive than apartment-type resources.

Housing influenced the relationship residents had with psychiatry. In residential accommodations, residents often must receive treatment to be able to stay, especially when these accommodations receive financing from hospitals. Apartment-type resources with or without support generally have no restrictions related to receiving treatment. However, some participants had a desire to maintain a relationship with psychiatry, and the ones who did not compensated by depending on other community mental health resources.

One problem that still needs to be addressed is that the participants from both categories felt isolated from “normal society.” The study’s discussion argued that defining the concept of integration as a process rather than a state would de-compartmentalize the social integration of individuals living with severe mental illness. De-institutionalization brought along challenges and adapted services need to address issues such as integration and normalization.

In other studies, little evidence was found to corroborate the superior efficacy of later models (e.g. supported housing) over others to promote recovery (39). However, housing stability has been found to be a strong predictor of reduced rates of shelter use, hospitalization, and homelessness, and has been found to be correlated to recovery (39, 40). In most of the research on housing, the housing retention rate is measured to access the efficacy of these models (40). We argue that more research using measures such as recovery (related to the mental health condition) would offer a broader picture of the efficacy of these different approaches.

## At Home/Chez Soi Program

When deinstitutionalization politics began and massive amounts of patients from mental asylums required housing, their first residences were not so different from hospital housing units based on the then dominating custodial and supporting housing models. These residences hosted nine individuals each, with 40 individuals per block and group treatment plans unseparated from housing and copied asylums’ operating: one bed per dorm, meals taken in groups, body hygiene, medication three times a day and leisure. Many authors (41–44) qualified these residences as caretaking that perpetuated the same problems that faced institutional housing (depersonalization, apathy, behavioral problems) without presenting the positive characteristics of these institutions (social contact, activities and programs, rehabilitation, and especially housing stability). *Housing First/Logement d’abord* is the antithesis of treatment first approaches, which was previously prioritized over housing. This model considers housing as a social right that cannot be conditional to following medical treatment or not consuming drugs.

*At home/Chez soi* was based on harm reduction and rehabilitation philosophies that put the person first. According to one of the project’s main researchers (45), *Housing First*, originating from New-York, seeks to give access to permanent and independent housing with support for homeless individuals with high to moderate needs in mental health. This support includes a multidisciplinary team that organizes intensive follow up in the community depending on the residents’ needs. This group of outreach workers was supported by a housing team that organized apartment visits and managed conflicts between the program participants and their neighborhood and landlords. Considering that some marginalized groups use up to 50% of their income for housing and to balance the insufficient funds offered by welfare, the project offers financial support as high as 70% of the housing costs. Outreach workers organize frequent visits and aim to develop the program participants’ autonomy. The project supports these participants with legal issues, with security concerns and crisis management, with rebuilding relationships with their families, and offers activities promoting social integration and social interactions in the community. The project was financed by Health Canada and was administered in five cities: Vancouver, Moncton, Toronto, Montreal, and Winnipeg. The goal was to examine the effectiveness of the *Housing First* approach (37), which values housing as a fundamental right and as a pathway to psychiatric rehabilitation (46). Program participants (homeless individuals with mental illness) were helped by being provided housing (notably by receiving rent supplements) prior to abstinence or being evaluated as “ready” for housing, while maintaining a consumer-driven approach. The overall results of this study were positive, as after 2 years 62% of the participants had been housed for 6 months or more (47). Four hundred sixty-nine individuals were recruited in the *At home/Chez soi* project, which included 285 participants in an *Housing First* experimental group receiving the model’s services and 184 in a control group receiving services as usual (48). Most participants in the experimental group reported appreciating the quality and the consistency of the support offered by this model (45). These participants mentioned that their housing helped them feel like they had a place in the world, to be recognized as individuals, and to develop their autonomy. Housing stability was higher in the experimental group than in the control. Six months before the end of the study, 60% of the experimental group participants were housed all the time compared to 31% in the control group, and 21% of the first group were not housed at all compared to 59% in the control group (48). In general, *Housing First* programs were also found to have an 80% housing retention rate even with individuals who were previously perceived as not “housing ready” (36). Consequently, less readmissions in psychiatric hospitals and incarcerations were reported. The participants in the *Housing First* group of the experiment were more likely to report improvements related to their mental health, community functioning, and positive social interactions (48).

Landlords play an important role in the accessibility to autonomous housing for individuals with mental health disorders. In this regard, MacLeod et al. (49) conducted a qualitative study on the experiences of landlords in the *At home/Chez soi* Canadian research project. Sixty-three interviews with landlords and housing management were conducted in the related qualitative study, in four cities (Moncton, 23; Toronto, 16; Montreal, 12; Winnipeg, 12). The context in which the program was administered varied considerably from one city to the other (see 49).

The authors mention the Landlord-Service Provider Forum model (50). Its goals are “(1) to clarify the responsibilities, rights, and roles of landlords, service providers, and tenants; (2) to facilitate communication and shared problem-solving; (3) to increase housing stability; (4) to retain cooperative landlords; and (5) to recruit new landlords and expand known housing stock.” (49; page 6). This model offers a possible avenue for offering support and education about individual rights and mental health to landlords in scattered-site housing. Bengtsson-Tops and Hansson's (51) qualitative study is cited, as it identifies three themes of the experiences of landlords with tenants with mental illness. The first theme was experiencing difficult circumstances related to the tenants’ mental illness and was perceived as time-consuming and problematic. The second theme was providing assistance, as landlords were helpful to the rehabilitation of the tenants by providing security and informal support. The final theme was that landlords felt like they did not have the resources to deal with the difficulties associated with housing tenants with mental illness. The previously mentioned Landlord-Service Provider Forum model might be useful for providing these resources (49).

*At home/Chez soi* and *Housing First/Logement d’abord* pilot projects revolutionized housing for marginalized groups, homeless individuals, and psychiatric patients in Canada and Quebec. These changes marked an evolution from the previously prevalent supportive housing to supported housing by offering more housing options that followed the model’s values. In the years following these projects, permanent housing with support gained considerable traction. MSSS’ Mental Health Action Plan reflects this ideologic change in its recommendations (18).

## Permanent Housing With Support

Permanent housing with support is not by any means a new concept in the field of housing studies. However, its widespread use and support from governmental agencies in Quebec is a rather recent development. As for many other discoveries, progress in housing for people with mental illness is achieved through trial and error and through the observation of other successful experiments in related fields. Permanent housing with support (PHS) is no exception to this rule. Temporary accommodations or housing, which were widespread after the deinstitutionalization of housing for people with mental illness, have been criticized for creating housing instability and limiting rehabilitation. Short term assessment of the problems associated with mental illness and homelessness jeopardizes the progress achieved in these programs once the supported individual leaves the program. Permanent housing with support, based on the supported housing model, counteracts these limitations by offering a community for these individuals in which to grow and develop autonomy while still receiving support when necessary. PHS combines housing with different types of support and intervention philosophies. For example, some PHS units offer entry with no prior conditions, while others prioritize harm reduction and have some prerequisites (40). PHS services are diverse in nature: scattered-site housing, housing units similar to low-cost housing (LCH) or community-managed apartments (52). Support is offered in a community-based setting or through home visits (53). These different types of PHS have been tested in the USA, in Europe, and in Australia notably. In America, PHS are mostly privatized and scattered throughout the community, including rent supplements and support of diverse intensity depending on the individual’s needs. In contrast, Australian PHS prioritize community units where marginalized individuals (homeless, people with mental illness, etc.) live together in their own apartments supervised by outreach workers (54).

In Quebec, government politics favor community-managed PHS or municipal LCH. Some studies have shown that private, social, and community-based PHS reduce the use of shelters (55, 56), hospitalizations (57) and incarcerations (58, 59), while increasing housing stability (40). In their research comparing single mothers living in temporary housing to ones living in permanent housing units, Letiecq et al. (60) found that mothers living in permanent housing had significantly more social interactions, maintained more relationships with their families and perceived that they had more available support than homeless mothers living in temporary housing did. These factors are especially crucial for rehabilitation. One study by Gentilet al. (61) found that the quality of life and social integration of homeless individuals were not significantly different between various PHS types. However, there is still a need for more research comparing different types of PHS services to identify their different effects on their users and to identify these users’ characteristics.

There are still many limitations concerning the implementation of PHS services. Leff et al. (40) have found limited evidence of the model’s superiority over other housing models. These authors suggest that different interventions might offer different advantages that might be more suitable for individuals with different needs. They suggest that an individualized approach to services might be more efficient than limiting these services to one approach or model. Another limitation related to the study of PHS services is the lack of consistent operationalization and variable application of its theoretical framework in practice (40).

Further research should be carried out to access the efficacy of this model compared to others. Focus should also be shifted toward the operationalization of the services offered in each types of housing analyzed. This shift would allow the identification of the specific elements leading to better outcomes.

For the sake of a metaphor, housing and rehabilitation could be represented as learning how to swim. The Custodial Model and similar models of housing could be trying to teach swimming by offering theory classes, the *At Home/Chez soi* project and autonomous apartments could be trying to teach swimming by throwing learners in the water (to a certain extent, depending on the offered support) and PHS could be teaching swimming by offering practical lessons with support.

Complementarily to these different housing models, there exists a dynamic network of community organizations that offer support to many different populations of marginalized individuals. According to Morin and Baillergeau (62), social housing with community support are non-profit, government subsidized housing accommodations where tenants are taught basic skills to look after their unit autonomously. Stable housing with affordable cost and long-term support seems to be the most efficient method to allow the social integration and rehabilitation of individuals with mental illness.

## Conclusion

In the last 50 years following the deinstitutionalization of psychiatric facilities organizing housing, the housing situation in Quebec fielded a wide array of different housing models. These models have evolved with its society and its values, but also benefitted from the experimentation on what works and what does not.

The problematic of homelessness, which affects many people with mental illness, dates back from the dawn of any social organization. Historically, self-reliance has been closely associated with the resources offered by owning property. But what becomes of the ones who do not own property? Since the creation of the Welfare State in the UK in the end of the 19^th^ century, government considers social protection a fundamental right and a basic aspect of living in a solidary, even democratic society. Projects such as *At Home/Chez soi*, helping homeless to get access to housing at reduced costs, are a demonstration of the implication of the government in the social security and social integration of its citizens. This implication is a testament to the humanistic values of our society as well as a bet on the benefits that come with the rehabilitation of marginalized individuals such as people with mental illness.

Many countries of the economic European community, including the UK, have housing benefits programs that reduce the gap between low income and the need for quality housing. These politics infer that housing is a social determinant of health, even more so than healthcare (63). As the WHO defines it: ‘Health is a state of complete physical, mental, and social well-being and not merely the absence of disease or infirmity.”

## Author Contributions

Both authors worked on research, writing, editing, and formatting (HD, JT-G).

## Conflict of Interest

The authors declare that the research was conducted in the absence of any commercial or financial relationships that could be construed as a potential conflict of interest.
